# Assessing the relationship between routine and schizophrenia symptoms with passively sensed measures of behavioral stability

**DOI:** 10.1038/s41537-020-00123-2

**Published:** 2020-11-23

**Authors:** Joy He-Yueya, Benjamin Buck, Andrew Campbell, Tanzeem Choudhury, John M. Kane, Dror Ben-Zeev, Tim Althoff

**Affiliations:** 1 Paul G. Allen School of Computer Science & Engineering, University of Washington, Seattle, USA; 2 Department of Psychiatry and Behavioral Sciences, University of Washington, Seattle, USA; 3 Department of Computer Science, Dartmouth College, Hanover, USA; 4 Cornell Tech, New York City, USA; 5 The Donald and Barbara Zucker School of Medicine at Hofstra/Northwell, East Garden City, USA

**Keywords:** Schizophrenia, Psychiatric disorders, Psychosis

## Abstract

Increased stability in one’s daily routine is associated with well-being in the general population and often a goal of behavioral interventions for people with serious mental illnesses like schizophrenia. Assessing behavioral stability has been limited in clinical research by the use of retrospective scales, which are susceptible to reporting biases and memory inaccuracies. Mobile passive sensors, which are less susceptible to these sources of error, have emerged as tools to assess behavioral patterns in a range of populations. The present study developed and examined a metric of behavioral stability from data generated by a passive sensing system carried by 61 individuals with schizophrenia for one year. This metric—the Stability Index—appeared orthogonal from existing measures drawn from passive sensors and matched the predictive performance of state-of-the-art features. Specifically, greater stability in social activity (e.g., calls and messages) were associated with lower symptoms, and greater stability in physical activity (e.g., being still) appeared associated with elevated symptoms. This study provides additional support for the predictive value of individualized over population-level data in psychiatric populations. The Stability Index offers also a promising tool for generating insights about the impact of behavioral stability in schizophrenia-spectrum disorders.

## Introduction

Individuals vary in the extent to which they consistently engage in the same patterns of behavior each day, i.e., routine stability. In the general population, a stable daily routine is linked with well-being^1^. This appears to be the case to an equal if not greater extent among individuals with schizophrenia-spectrum disorders (SSDs). Individuals with SSDs who consistently engage in activities that typically occur in a routine—e.g., employment^2^, education^3^, healthy sleep^4^, social connections^5^, and physical activity^6^—enjoy an array of physical and mental health benefits. Further, many psychosocial interventions that promote wellness routines—e.g., behavioral activation and scheduling, Illness Management and Recovery, or Wellness Recovery Action Plan—reduce depressive^7^ and psychotic symptoms^8^ and improve functioning^9^. Taken together, this evidence suggests that adherence to one’s routine could be indicative of continued stability, while deviation could indicate risk for worsening symptoms.

The study of behavioral stability has been limited by the use of retrospective scales, which are common in clinical research. These measures require respondents to provide estimates of the amount and frequency of behaviors over weeks or months. Such estimates are insufficiently granular to assess behavioral stability. They are also susceptible to memory inaccuracies^10^, or—if administered by an interviewer—interpretive errors^11^. Further, completing assessments in clinical research settings could lead to minimization, over-reporting, or unconsciously responding to demand characteristics embedded in the assessment context^12^.

Emerging technologies can mitigate these sources of error. Two key innovations may provide the opportunity to assess behavioral stability and its association with psychiatric symptoms, ecological momentary assessment (EMA^13^) and passive sensing. EMA, which involves the direct administration of brief measures in respondents’ day-to-day lives, appears feasible to administer via mobile devices and acceptable to individuals with chronic^14–16^ and early SSDs^17,18^. Passive mobile sensing systems build on EMA by gathering data to estimate the frequency and intensity of behaviors. Cognitive, affective, and behavioral data collected with passive sensors and EMA may signal changes in symptoms and functioning. Such predictive models have been tested in major depressive disorder^19–21^, bipolar disorder^22,23^, schizophrenia^24,25^, and older adults with depression^26^. These technologies provide the capacity to examine specific questions about the relationships between structured routine and symptoms.

A recent study^27^ demonstrated that less routine in terms of the regularity of the locations people visited was associated with greater severity of psychiatric symptoms among individuals with schizophrenia but not healthy controls. Previous studies^19,28^ also reported that stability of daily movement was the feature most highly associated with depressive symptoms across multiple behavioral features. Other studies using EMA and sensing in schizophrenia support the more idiographic use of mobile data^29^, since behavioral patterns that precede increases in symptoms are often individual-specific (i.e., “relapse signatures”^30^). These studies suggest that deviations from routine could directly indicate increased psychiatric risk, and understanding the relationship between routine and schizophrenia symptoms may offer clinically relevant and actionable insights for early detection of behavioral changes and symptom exacerbation among people with SSDs. However, many of the existing measures of routine^19,27,28^ are restricted to mobility-related behaviors and do not generalize to other behaviors (e.g., phone calls and sleep). Other methods such as the regularity index^31^ generalize to other behaviors, but are sensitive to small shifts in routines (e.g., a few minutes across the hour mark) because they are restricted to hourly aggregates of behaviors.

We extend previous work by proposing a technique that generalizes to a variety of passively sensed behaviors. Our technique is less sensitive to small shifts in routines and works for any time granularity because it compares cumulative distributions of activities that can be used with a variety of time scales. To demonstrate the utility of these behavioral stability features, we use the Crosscheck data set^25,30,32^, which was previously collected by our team using a multi-modal mobile assessment system in a sample of individuals with schizophrenia for 12 months. Concretely, in this secondary analysis, we demonstrate the utility of our technique by answering five research questions:

1. How can passively sensed behavioral stability can be quantified?
2. Are behavioral stability features associated with symptoms and dysfunction in schizophrenia?
3. Is behavioral stability predictive of symptoms and dysfunction?
4. Do existing data from other individuals help in predicting symptoms?
5. Can behavioral stability features be used to predict symptoms and dysfunction in the near future?

## Results

We proposed a metric, the Stability Index, to quantify behavioral stability (see Section “Data analytic plan”). We calculated the Stability Index for each activity listed in Table 1. A higher Stability Index indicates less difference and more stability (see examples in Fig. 4). The severity of psychiatric symptoms (see Table 3) is measured by the EMA score (see Section “Ecological momentary assessment”), which ranges from −15 to 15. A higher score suggests greater symptom severity and poorer functioning.

**Table 1:** Features used for prediction.

Number of incoming messages
Number of outgoing messages
Call periods
Phone unlocked periods
Conversation periods
Ambient light intensity
Ambient sound volume
Ambient voice sounds periods
Ambient non-voice sounds periods
Ambient silence periods
Sleep periods
On-bike periods
Walk periods
In-vehicle periods
Tilting periods
Still periods
Unknown activity periods

### Correlations between behavioral stability and symptom severity

Results (see Table 2) show that psychiatric symptom severity was positively correlated with stability in when the participants were still (i.e., non-moving) (*r* = 0.265, *p* < 0.001) and when the participants were surrounded by non-voice sounds (e.g., in a noisy busy place) (*r* = 0.176, *p* < 0.001)‚ suggesting that more stability in when participants were still or surrounded by noisy sounds was associated with greater symptom severity and poorer functioning. Moreover, psychiatric symptom severity was negatively correlated with stability in the number of text messages the participants sent out (*r* = −0.192, *p* < 0.001), when the participants had phone calls (*r* = −0.159, *p* < 0.001), when the participants unlocked their phones (*r* = −0.153, *p* < 0.01), and the number of text messages they received (*r* = −0.147, *p* < 0.01). These correlations indicate that greater stability in when participants sent messages, had phone calls, unlocked their phones, or received messages was associated with lower symptom severity and better functioning. Across all of these behaviors, the magnitude of the correlations in our data set matched that of previous work focused on the regular recurrence of GPS locations^27^.

**Table 2:** Correlations between the Stability Index and psychiatric symptom severity.

Stability Index of sensed behaviors	*r*
Stability of still periods	0.265^***^
Stability of ambient non-voice sounds periods	0.176^***^
Stability of tilting periods	0.110
Stability of unknown activity periods	0.105
Stability of ambient sound volume	0.084
Stability of walk periods	0.037
Stability of sleep periods	0.022
Stability of on-bike periods	0.019
Stability of in-vehicle periods	0.009
Stability of ambient silence periods	0.006
Stability of ambient voice sounds periods	−0.014
Stability of conversation periods	−0.022
Stability of ambient light intensity	−0.053
Stability of number of incoming messages	−0.147^**^
Stability of phone unlocked periods	−0.153^**^
Stability of call periods	−0.159^***^
Stability of number of outgoing messages	−0.192^***^

These correlations hold across the entire one-year study period and for multiple participants (*n* = 13).

^**^p < 0.01, ^***^p < 0.001.

### Prediction of symptom severity

For each individual, we trained and tested a personalized model (i.e., a model that used only that individual’s data for training) on different sets of features: previous EMA score (7 days prior), mean and standard deviation of the amount of each behavior across a 2-week period (i.e., current state-of-the-art features^32^), the Stability Index, and the Stability Index combined with mean and standard deviation of each behavior. The performance of the “individual-only” models was evaluated by calculating the mean absolute error (MAE) of predictions.

**Fig. 1:**
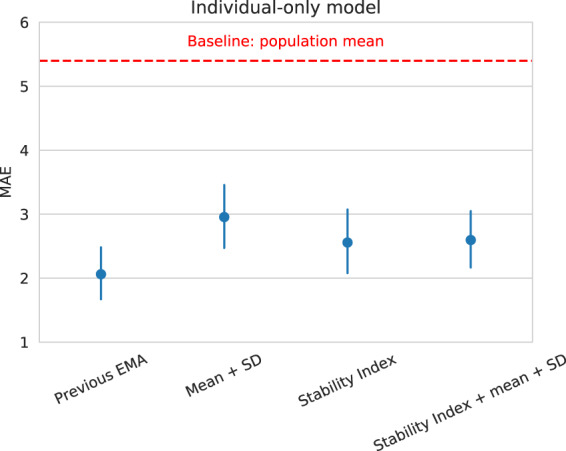
Comparison of Stability Index-based predictions of symptom severity to current state-of-the-art features. The error bars indicate 95% bootstrapped confidence intervals. Previous EMA score (7 days prior) performed the best. The model trained on only the Stability Index achieved nearly identical performance. This performance was much better than the baseline model, which always predicted the population mean (indicated by the dashed red line). In addition, the model trained on only the Stability Index performed equally well as the current state-of-the-art model trained on the mean and standard deviations of the amount of each behavior^32,38,39^.

Results (see Fig. 1) show that the best performing model was trained on the previous EMA score (MAE = 2.061), followed by the Stability Index (MAE = 2.556), the Stability Index combined with the mean and standard deviation of the amount of each behavior (MAE = 2.595), and the mean and standard deviation model (MAE = 2.954). From a practical perspective, the errors of the “previous EMA” model and the “Stability Index” model were close (2.061 vs. 2.556) and statistically indistinguishable (*p* = 0.055; two-sided Wilcoxon *T*-test). Although our behavioral features did not improve performance over the previous EMA, in practice our behavioral features are still useful whenever EMA scores are not available. In our study, EMA scores were missing about 37% of the time. Thus, an EMA score from 7 days ago was often not available to make a prediction. Notably, the Stability Index matched the performance of current state-of-the-art features (i.e., mean and SD) ^32^, and improved performance over the baseline model that always predicts the population mean of the EMA score (MAE = 5.396).

### Prediction of symptom severity using population data

**Fig. 2:**
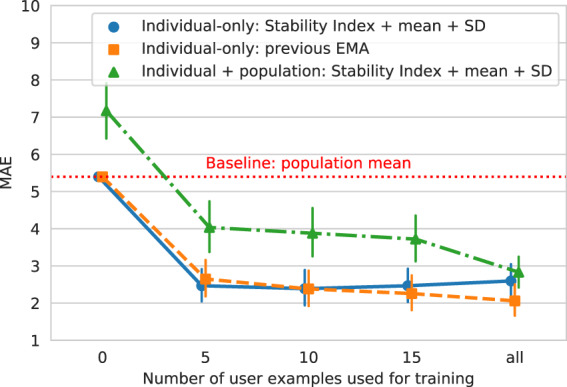
Comparison of the “individual-only” model and the “individual + population” model using varying numbers of user (i.e., within-person) examples for training. For the “individual-only” model using zero user examples, we obviously did not have any data for training, so we used the population mean baseline as a fallback prediction. The error bars indicate 95% bootstrapped confidence intervals. This shows that the “individual-only” model trained on the Stability Index, mean, and SD (represented by the solid blue line) was statistically indistinguishable from the model trained on previous EMA (the dashed yellow line), demonstrating that these behavioral features were able to predict as well as the previous EMA, which is a strong baseline as shown in Section “Prediction of symptom severity”. This also demonstrates that no matter how little data you have of the individual, adding additional data from other individuals never helped in this prediction task.

To examine whether data points from other individuals help in predicting symptom severity, we compared the “individual-only” model with the “individual + population” model trained on data from both the individual and the rest of the population. As shown in Fig. 2, at no point did the “individual + population” model outperform the “individual-only” model. No matter how little data were used to train the “individual” model, adding data from other people never helped in prediction. Notably, the model using only these population data performed worse than the baseline model (i.e., predicting with the mean of the EMA score). This establishes that knowing any data from other individuals is not helpful for this prediction task due to high variability among subjects (see Supplementary Fig. 1). On the other hand, only a few data points are needed from one individual to make a good prediction about their symptoms. In fact, using only five data points, which span less than a month, achieved the best performance of the models tested. This means that additional data points that are further back in time may not help in prediction, possibly due to time-varying nature of symptoms^32^. Thus, using passive sensors, reasonably accurate predictions (MAE = 2.468) can be made with a month of individual data.

### Prediction of future symptom severity

**Fig. 3:**
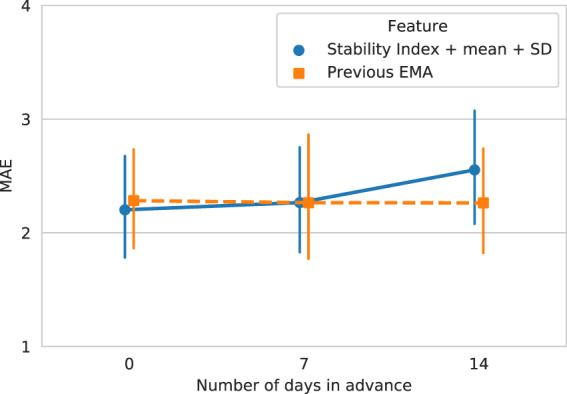
Comparison of the “individual-only” models trained on data from 0, 7, and 14 days prior. The error bars indicate 95% bootstrapped confidence intervals. The solid blue line indicates results of the model trained on the full set of behavioral features. The MAE did not increase significantly as we tried to predict more in advance. The dashed yellow line shows results of the model trained on the previous EMA score. There is also no significant difference between the model trained on all behavioral features and the model trained on the previous EMA score, which is a strong baseline as shown in Section “Prediction of symptom severity”. This demonstrates that the Stability Index features can help predict symptom severity up to 14 days in advance.

As shown in Fig. 3, the performance of the model predicting current EMA scores (MAE = 2.202) was statistically identical to the models predicting EMA scores 7 days in advance (MAE = 2.266; two-sided Wilcoxon *T*-test: *p* = 0.540) and predicting EMA scores 14 days in advance (MAE = 2.553; two-sided Wilcoxon *T*-test: *p* = 0.112). Data sets were kept comparable by removing the EMA data points that were not in the 14-day data set from the 0-day and 7-day data sets (see Section “Data analytic plan”). We predicted only 14 days in advance because going further would have drastically reduced data set size, as most participants did not consistently log at least 7 days of data over each 14-day period across the entire study period, and we would also need to ignore the earliest EMA data points for which there was no previous data to compute features from (see details in Section “Data analytic plan”). Thus, while we could still predict accurately up to 2 weeks in advance, we were not able to establish longer-term predictions because of this reduced data set size.

## Discussion

Stability of routine has been suggested as an intervention target to assist in the recovery of individuals with SSDs, but there are few consistent definitions of routine that can be efficiently assessed in real-time and real place. Our group developed a stability index using data collected by passive sensors in a sample of over 60 individuals with schizophrenia. The Stability Index derived here provides a metric to assess the extent to which an individual adheres to a stable routine. The present study demonstrates that this metric adds value when predicting psychiatric symptoms above and beyond other population-level parameters. The Stability Index matched the predictive performance of current state-of-the-art features^32^, while being orthogonal to these features and being interpretable and relevant to construct of routine. This suggests that our measure of stability—which has been proposed as an important metric to quantify in previous research—is a contribution to assessments of behavior and functioning in schizophrenia. It may be the case that in larger samples over longer periods of time, this metric could further improve predictions of future symptoms and functioning.

There is a growing body of support for the use of passive sensors in assessing clinical status for a number of psychiatric conditions^25,30^. However, a key persisting question in the use of passive sensors in psychiatry pertains to the utility of population-level vs. individual-referenced data^29^. This study suggests that referencing on the individual level may provide more insightful psychiatric predictions than examining raw sensor values that reference on population values. In our data, only within-subject passive sensor data improved predictions of psychiatric symptoms.

Relationships between the Stability Index and psychiatric symptoms appear informative about indicators of functioning with regard to specific symptoms. Stability in two sensors that assess movement and the surrounding context encountered by the participant—stillness and nearby sounds—was associated with greater psychiatric symptoms, while in several social-oriented sensors—including phone calls and SMS messages—greater stability was associated with lower symptoms and better functioning. It’s possible that this indicates that stability in social contact is a sign of improved symptoms or functioning. This is consistent with our group’s previous work that demonstrated smartphone-based social behavior appeared to change in the period preceding relapse^24^. Stability in the sensors that detect movement and surrounding context, on the other hand, could indicate inactivity, social avoidance, or sedentary behavior. Active days for the average person may involve more irregular bursts in activity, for example, when an individual interacts with others at work, runs errands, or engages in physical activity. As a result, for these activities, greater instability could in fact be indicative of improved functioning.

Finally, while there were few relationships between specific passive sensor variables and psychiatric symptoms, quantifying the change in individual symptoms enabled accurate predictions of symptoms using only few (i.e., five) data points. The Stability Index predicted future (e.g., 7, 14 days) symptom severity, as predictions using data from 0, 7, and 14 days prior had relatively equal accuracy in prediction. This indicates that mobile assessment systems that incorporate passive sensors could begin to make reasonable predictions about users’ symptoms only after a relatively brief training period.

This study is not without limitations. First, models were all trained on a relatively small sample of individuals with SSDs, and assessments of symptoms were reported only on selected days. At most, participants reported symptom levels three days per week. This lack of data may have contributed to reduced predictive power, as evidenced by large but non-significant raw value differences in accuracy of the Stability Index model and the state-of-the-art model (using mean and SD)^32^. However, any increases in frequency of prompts has a trade-off in increasing participant burden, particularly for a one-year study period. As larger data sets become available with the expansion of these methods, future studies should examine with greater power the additive predictive value of stability estimates above and beyond such baseline models. Last, the symptom assessment deployed in this study was limited by the brevity required by EMA. Such a measure, though face valid, lacks extensive psychometric validation at present.

The Stability Index examined here quantifies a measure of a domain that has been a target of clinical intervention but has lacked tools for accurate and efficient assessment. The Stability Index appears orthogonal from existing metrics drawn from passive sensors and generates insights about the impact of stability in a range of behaviors in SSDs. This index offers a promising tool for assessing changes in behavior over time and predicting future symptom levels.

## Methods

Data in this study were drawn from a randomized trial of mHealth monitoring intended to reduce psychiatric relapse in SSDs. This trial was approved by the IRBs of Dartmouth College (#24356) and Northwell Health/Long Island Jewish Medical Center (#14-100B) and registered as a clinical trial (#NCT01952041).

### Participants

Participants included 61 (*n* = 61) adults with a schizophrenia-spectrum disorder and a recent (within past 12 months) significant psychiatric event, including either a psychiatric inpatient or daytime hospitalization, psychiatric ER visit, or outpatient crisis management. Exclusion criteria were: (1) sensory or physical impairments that would interfere with the use of a smartphone (determined via screening in vivo testing), (2) a <6th grade reading level (per the Wide Range Achievement Test^33^), or (3) lacking competency to consent to participate in research.

### Procedure

Full descriptions of the study software^25,30,32^ and other studies examining CrossCheck data^24,34^ are available elsewhere. Participants were recruited from a large psychiatric hospital in New York. Clinicians were asked by the research team to provide these prospective participants with a study description and post flyers. Study staff also reviewed electronic health records for potentially eligible clients to approach. The research team oriented potential participants to the study when these prospective participants authorized clinicians to share their contact information. After completion of written informed consent, participants were randomized into either: (1) the intervention group (i.e., with access to the CrossCheck system with as needed follow-up support), or (2) the treatment as usual group.

Data in this report are from participants in the intervention group (i.e., CrossCheck condition). All participants in this condition were asked to carry a Samsung Galaxy S5 Android smartphone with CrossCheck pre-installed with them for 12 months. Three days per week, CrossCheck prompted participants to complete a brief self-report scale; at the same time, it collected data from passive sensors already installed on devices in the background of the user’s otherwise routine device use. Table 1 shows a full list of behaviors used for prediction.

### Ecological momentary assessment

CrossCheck prompted participants to complete a 10-item self-report (EMA) questionnaire each Monday, Wednesday, and Friday during the study period. This questionnaire began with the prompt, “Just checking in to see how you’ve been doing over the last few days”. Table 3 shows the full list of EMA items; response options ranged from 0 (not at all) to 3 (extremely). For this analysis, we calculated overall EMA score as the sum of all negative items minus the sum of all positive items. This score ranges from −15 to 15, with a higher value suggesting greater symptom severity and poorer functioning.

**Table 3:** EMA items.

Prompt: Just checking in to see how you’ve been doing over the last few days.
1. Have you been bothered by *voices*?
2. Have you been *seeing things* other people can’t see?
3. Have you been feeling *stressed*?
4. Have you been worried about people trying to *harm* you?
5. Have you been *depressed*?
6. Have you been feeling *calm*?
7. Have you been *social*?
8. Have you been *sleeping* well?
9. Have you been able to *think* clearly?
10. Have you been *hopeful* about the future?

Questions 1–5 are negative items, and questions 6–10 are positive items. Response options: 0—not at all; 1—a little; 2—moderately; 3—extremely.

### Multimodal behavioral sensing measures

#### Physical activity

CrossCheck assessed physical activity using Google Activity Recognition Application Programming. Every 10 s, CrossCheck generated a rating of which activity the participant engaged in or every 30 min when the device was held still. As shown in Table 4, participants in the study spend an average of 18.2 min walking, 28.6 min being in vehicle, and 467.4 min being non-moving per day.

**Table 4:** Feature summary.

Behaviors	Mean	SD	Min	Median	Max
Number of incoming messages	3.4	6.8	0.0	0.9	36.7
Number of outgoing messages	3.4	8.0	0.0	0.4	49.4
Call periods (min)	10.9	19.2	0.0	5.0	136.2
Phone unlocked periods (min)	98.2	129.8	0.1	52.6	512.7
Conversation periods (min)	117.1	81.3	3.2	111.8	384.8
Ambient voice sounds periods (min)	54.5	51.7	0.2	53.0	198.7
Ambient non-voice sounds periods (min)	144.1	155.8	1.0	119.2	763.5
Ambient silence periods (min)	933.3	453.3	13.9	1150.1	1430.7
Sleep periods (min)	630.6	336.5	9.0	650.8	1360.7
On-bike periods (min)	1.2	1.3	0.0	0.7	6.0
Walk periods (min)	18.2	19.6	0.0	12.5	78.2
In-vehicle periods (min)	28.6	24.5	0.0	23.7	90.5
Tilting periods (min)	42.9	34.0	0.2	38.0	134.0
Still periods (min)	467.4	193.4	47.4	469.3	778.0
Unknown activity periods (min)	370.2	172.3	72.0	370.8	1378.4

These are statistical measures of all participants’ average daily amount of each behavior. For example, the average number of incoming messages received per day is 3.4 across multiple participants (*n* = 59).

#### Speech frequency and duration

CrossCheck passively assessed (via the Smartphone microphone) the amount of time during which speech was present or near to the device, allowing for quantification of speech frequency (the number of discrete episodes during which the device detected speech) and speech duration (the summed length of these episodes over the course of a day). Table 4 shows that participants in the study spend an average of 54.5 min being around human voice sounds, 144.1 min being in a noisy environment, and 933.3 min being in a quiet environment per day.

#### Device use measures

CrossCheck passively logged the number of SMS text messages sent and received as well as the number and duration of phone calls placed and received. Table 4 shows that participants in the study sends an average of 3.4 messages and have an average of 10.9 min of phone calls per day.

### Data analytic plan

#### Data filtering

Before calculating study variables, we filtered data to increase data quality. Consistent with previous work in our group^25^, we included only periods wherein 7 “good data” days occurred during the previous 14-day period, with “good data” days defined as those in which more than 19 h of sensing data were collected during that day. In order to conduct the experiments described in the section “Prediction of symptom severity using population data”, we needed at least 15 data points from each individual to train our models, and we chose the 10 most recent data points for each individual as the test set. Since we needed at least 25 data points from each individual, we restricted further analyses to the 13 participants with more than 25 EMA responses over the entire study period.

#### Calculating behavioral stability

Using the remaining data, we calculated the Stability Index. For a given activity (see Table 1), we calculated the distance function for the activity distributions on two different days, represented by two normalized cumulative sum functions. We then used the median distance function out of all the distance functions for all pairs of days within a 14-day period (preceding an EMA response) to characterize the degree to which behavioral patterns varied across that period. We next defined the Stability Index to be the inverse of the median distance function. A higher Stability Index indicates a less varying routine over time and thus more stability. Instead of computing the distance function for all pairs of days, we could have considered comparing only consecutive pairs of days. However, this approach would not capture behavioral stability for people who follow a routine but not consecutive schedule. For comparison, we also computed the mean and standard deviation of each sensor variable over the 14-day period to determine the predictive validity of our Stability Index metric.

To examine whether passively sensed behaviors can be used to predict symptoms up to 2 weeks in advance, we computed a data set for predicting 7 days in advance and 14 days in advance, respectively, by calculating the features over a 14-day period that is 7 days or 14 days prior to an EMA response. Compared to the data set used for predicting 0 days in advance, the data sets used for predicting 7 or 14 days in advance lose the EMA data points given over the earliest 7-day or 14-day period by each participant (for which no previous sensing data are available). There was an additional loss of data because most participants did not consistently log at least 7 days of data over each 14-day period across the entire study period (see our filtering method described above). To keep the data sets used in the section “Prediction of future symptom severity” comparable, we removed the EMA data points that were in the data set for predicting 0 days in advance but were missing in the data sets for predicting 7 or 14 days in advance.

The following provides a formal definition of the Stability Index.

Let *x* denote the *x*th minute of a day. Let ${A}_{b}^{d}(x)$ be the amount of a given behavior *b* done by a user at minute *x* of day *d*. Let *M* = 1440 be the total number of minutes of a day. Then, we define the normalized cumulative sum function ${C}_{b}^{d}(x)$ as

1 $${C}_{b}^{d}(x)=\frac{\sum_{i = 1}^{x}{A}_{b}^{d}(i)}{\sum_{i = 1}^{M}{A}_{b}^{d}(i)}$$

For example, ${C}_{\mathrm{call}\,}^{d}(600)=0.5$ means that this particular patient did 50% of all of their phone calls by the 600th minute of the day, or 10 a.m.

Then, given ${C}_{b}^{{d}_{1}}(x)$ and ${C}_{b}^{{d}_{2}}(x)$ for a pair of days (*d*_1_, *d*_2_), we define the distance function *D*_*b*_(*d*_1_, *d*_2_) as the average distance between two normalized cumulative sum functions:

2 $${D}_{b}({d}_{1},{d}_{2})=\frac{1}{M}\sum_{i = 1}^{M}| {C}_{b}^{{d}_{1}}(i)-{C}_{b}^{{d}_{2}}(i)|$$

Now we can define the Stability Index SI_*b*_(*P*) of a period of multiple days *P* = {*d*_1_, . . ., *d*_*N*_}, where *N* is the number of days in that period, as

3 $${\mathrm {S{I}}}_{b}(P)=1-{\rm{median}}(\{{D}_{b}(i,j)| i,j\in P,i \, \ne \, j\})$$

**Fig. 4:**
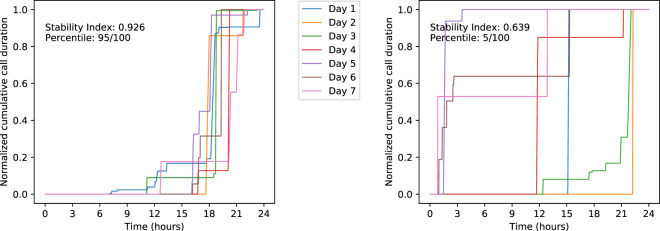
Comparison of a more stable behavioral pattern (on the left) to a less stable behavioral pattern (on the right). The left one has a higher Stability Index than the right one (0.926 vs. 0.639).

The Stability Index ranges between 0 and 1, with higher values indicating greater stability. For example, Fig. 4 compares the phone call distribution of a particular patient over two different weeks, one with a higher Stability Index (SI = 0.926, 95/100th percentile) and the other with a lower one (SI = 0.639, 5/100th percentile).

Instead of the normalized cumulative sum function ${C}_{b}^{d}(x)$, one could consider the absolute cumulative sum function without being normalized:

4 $${C}_{b}^{{\prime d}\,}(x)=\sum_{i = 1}^{x}{A}_{b}^{d}(i)$$

However, we find that the absolute Stability Index does not capture more information than the mean of the amount of behavior does. Supplementary Table 1 shows that the absolute Stability Index is extremely highly correlated with the mean (average *r* = 0.645), suggesting that the absolute Stability Index shows more about how much one engages in a certain activity than about the routine. In contrast, the normalized Stability Index is less correlated with the mean (average *r* = 0.304), which indicates that the normalized Stability Index captures information more orthogonal to the mean.

One could also consider using the raw activity distribution to compute the distance function. However, it is less reflective of behavioral stability than the normalized cumulative sum functions (Supplementary Fig. 2). For example, if a person’s schedules on two different days are almost the same but simply slightly shifted by a few minutes across the hour mark, then these days are not considered similar when examined hour by hour. Previously proposed methods such as the regularity index^31^ suffer from the same issue. Our behavioral Stability Index that is based on the cumulative activity distribution does not rely on aggregation of behaviors at a specific timescale and overcomes this problem.

#### Experiment details

First, to explore the relationship of the Stability Index to symptoms and dysfunction, we examined Pearson correlation coefficients (Table 2). A Bonferroni correction^35^ was applied on the *p*-values to reduce Type I Error rate given the high number of computed correlations. Correlations reported in Table 2 hold across the entire study period and for all participants (*n* = 13) left after filtering (see Section “Data analytic plan”). To determine whether these correlations are due to missing data, we also examined correlations obtained from requiring at least 8–14 “good data” days during the 14-day period. The results were qualitatively the same (see Supplementary Table 2). To examine how our Stability Index works with lower volume data, we also computed the Stability Index over a period of 7 days and investigated data availability requirement of 4, 5, 6, or 7 out of 7 days. The results show that even including people who only had 4 out of 7 days of sensing data available, we achieve significant correlations between our Stability Index and EMA score (see Supplementary Table 3).

Second, to examine predictive models, we identified the 10 most recent data points for each individual in the study period as the test set, and we varied whether predictive models used the 5, 10, or 15 previous data points preceding the test period in subsequent models. We used gradient boosted regression trees (GBRT)^36,37^ to predict EMA scores. GBRT is a model that uses an ensemble of weak regression trees to make predictions. It builds regression trees sequentially, and each regression tree estimator tries to reduce the bias of the previously combined estimators. The number of trees, maximum depth of the trees, and learning rate are three important hyper-parameters of the model. We set these hyper-parameters based on 10-fold cross validation using the training data. We tried numbers from 5 to 1000 for the number of trees, from 2 to 9 for the maximum depth of the trees, and from 0.01 to 0.3 for the learning rate. For similar choices of hyper-parameters, we found the predictive performance to be stable, but we chose a fixed set of hyper-parameters for all our experiments. The number of trees was 300; the maximum depth of the tree explored was 3; and the learning rate was 0.17. We trained the models on different sets of features: previous EMA score (7 days prior), mean and standard deviation of the amount of each behavior, the Stability Index, and the Stability Index combined with mean and standard deviation of each behavior. We then evaluated the performance of the models by calculating the MAE of our predictions. To determine the value of population data in predicting symptoms, we added an extra binary feature for the “individual + population” models to indicate whether a given example was from the same individual involved in this prediction, thus quantifying the added value of individual data above and beyond the full population data set.

### Reporting summary

Further information on research design is available in the Nature Research Reporting Summary linked to this article.

## Supplementary information

Supplementary Information

Reporting Summary

## Data Availability

The data that support the findings of this study are publicly available at: https://www.mh4mh.org/eureka-data.

## Code availability

The code that supports the findings of this study is publically available at: https://github.com/behavioral-data/stability-index.
