# Supplementary material for: Assessing the relationship between routine and schizophrenia symptoms with passively sensed measures of behavioral stability
Source: NPJ Schizophr. 2020 Nov 23;6:35. doi: 10.1038/s41537-020-00123-2 (PMC7683525; doi:10.1038/s41537-020-00123-2)
Supplement: Supplementary file 1 — Supplementary Information [file 41537_2020_123_MOESM1_ESM.pdf]

Supplementary Information

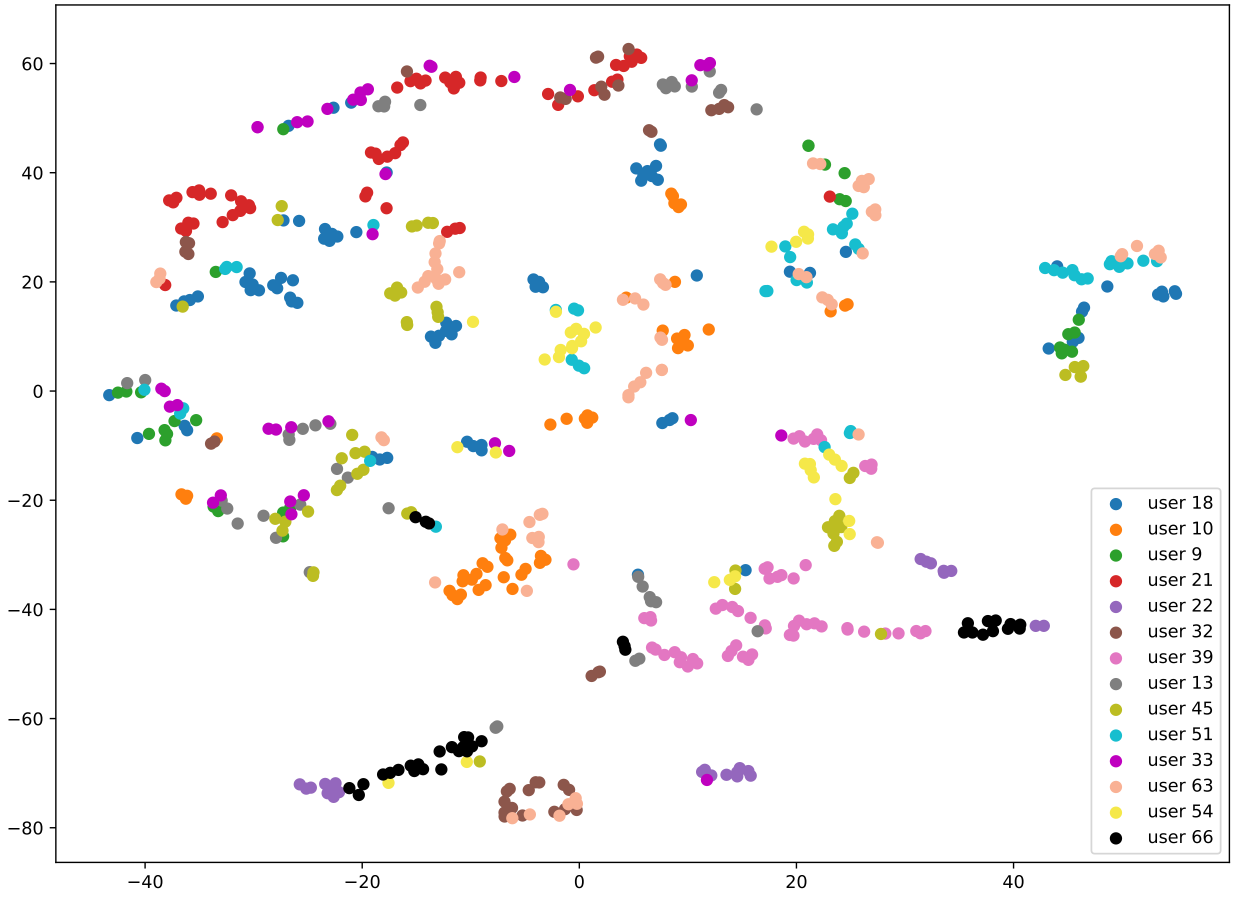

Supplementary Figure 1: Feature visualization using t-SNE (color coded by user ID). Each point represents a set of behavioral features computed over a two-week period preceding an EMA response. Participants tend to be more similar to themselves than to others. For some participants, there are no data points from other participants in their vicinity, which suggests that behavioral patterns differ from individual to individual. In fact, our analysis in Section "Prediction of symptom severity using population data" demonstrates that data from other individuals does not help with the prediction task.

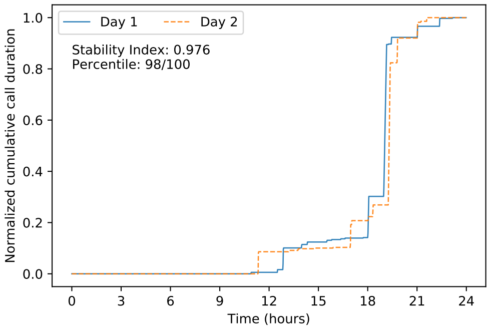

(a) The normalized cumulative sum functions of two different days

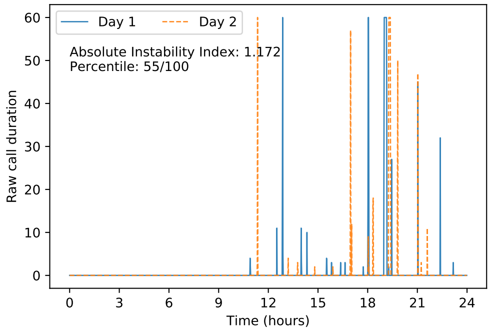

(b) The raw duration distributions of two different days

Supplementary Figure 2: Comparison of the normalized cumulative sum function to the raw distribution. The normalized cumulative sum function (a) can capture behavioral stability better than the raw distribution (b). When activity distributions for two days are almost identical but slightly shifted by a few minutes, the normalized cumulative sum function still suggests high stability (Percentile: 98/100), while the raw distribution suggests low stability (Percentile: 55/100).

Features	Normalized	Absolute
Stability of number of outgoing messages	0.362***	0.953***
Stability of number of incoming messages	0.387***	0.944***
Stability of call periods	0.231***	0.917***
Stability of phone unlocked periods	0.334***	0.839***
Stability of conversation periods	0.181***	0.739***
Stability of ambient light intensity	0.278***	0.925***
Stability of ambient sound volume	0.084	0.749***
Stability of ambient voice sounds periods	0.029	0.806***
Stability of ambient non-voice sounds periods	0.668***	0.343***
Stability of ambient silence periods	0.919***	0.326***
Stability of sleep periods	0.201***	0.124*
Stability of on-bike periods	−0.043	0.777***
Stability of walk periods	−0.025	0.870***
Stability of in-vehicle periods	0.265***	0.762***
Stability of tilting periods	0.424***	0.775***
Stability of still periods	0.100	0.270***
Stability of unknown activity periods	0.778***	−0.146**

Notes: $^{*}p < .05$, $^{**}p < .01$, $^{***}p < .001$

Supplementary Table 1: Comparison of correlations between the normalized Stability Index and the mean amount of the behavior to correlations between the absolute Stability Index and the mean. These correlations hold across the entire one-year study period and for multiple participants (n = 13). The absolute feature is highly correlated with the mean amount of the behavior (average $r = 0.645$), while the normalized Stability Index, a more orthogonal feature, is less associated with the mean (average $r = 0.304$).

**Stability Index of sensed behaviors**	**7 days**	**8 days**	**9 days**	**10 days**	**11 days**
Stability of still periods	0.265***	0.271***	0.268***	0.274***	0.297**
Stability of ambient non-voice sounds periods	0.176***	0.190***	0.161*	0.160	0.295**
Stability of tilting periods	0.110	0.113	0.137	0.147	0.196
Stability of unknown activity periods	0.105	0.122	0.077	0.043	0.167
Stability of ambient sound volume	0.084	0.085	0.087	0.029	0.103
Stability of walk periods	0.037	0.024	0.016	−0.041	−0.018
Stability of sleep periods	0.022	0.010	0.003	−0.064	−0.077
Stability of on-bike periods	0.019	−0.024	−0.027	−0.073	−0.137
Stability of in-vehicle periods	0.009	−0.001	−0.014	−0.067	−0.071
Stability of ambient silence periods	0.006	0.018	0.026	0.075	−0.131
Stability of ambient voice sounds periods	−0.014	−0.029	−0.015	−0.083	0.003
Stability of conversation periods	−0.022	−0.037	−0.040	−0.098	−0.033
Stability of ambient light intensity	−0.053	−0.052	−0.042	−0.085	−0.057
Stability of number of incoming messages	−0.147**	−0.190***	−0.247***	−0.272***	−0.272**
Stability of phone unlocked periods	−0.153**	−0.184***	−0.225***	−0.308***	−0.335***
Stability of call periods	−0.159***	−0.177***	−0.230***	−0.294***	−0.333***
Stability of number of outgoing messages	−0.192***	−0.234***	−0.300***	−0.340***	−0.428***

Notes: $^{*}p < .05$, $^{**}p < .01$, $^{***}p < .001$

Supplementary Table 2: Correlations between the Stability Index and psychiatric symptom severity if computing the Stability Index over a 14-day window and requiring at least 7, 8, 9, 10, or 11 "good data" days. These correlations hold across the entire one-year study period and for multiple participants (n $\geq$ 12). Table 2 in the main article was duplicated (the "7 days" column) for comparison. This table demonstrates that even including people who only had 50% of tracking (i.e. with 7 out of 14 days of sensing data available), our results are still qualitatively the same as those obtained by requiring more days of data (from 8/14 to 11/14). The correlation coefficients for these different data requirements are highly similar. We did not include correlations for 12–14 days because we would have much fewer participants (n < 12) with more rigorous filtering.

Stability Index of sensed behaviors	4 days	5 days	6 days	7 days
Stability of still periods	0.245***	0.263***	0.367***	0.483**
Stability of ambient non-voice sounds periods	0.159***	0.159*	0.290***	0.343
Stability of tilting periods	0.131**	0.147*	0.134	0.181
Stability of unknown activity periods	0.127*	0.080	0.160	0.216
Stability of ambient sound volume	0.102	0.090	0.043	0.050
Stability of walk periods	0.057	0.003	−0.118	−0.176
Stability of sleep periods	0.080	0.041	−0.086	−0.172
Stability of on-bike periods	−0.002	−0.037	−0.138	−0.384
Stability of in-vehicle periods	0.046	0.025	−0.085	0.000
Stability of ambient silence periods	−0.022	0.005	−0.012	−0.253
Stability of ambient voice sounds periods	0.038	−0.006	−0.107	−0.144
Stability of conversation periods	0.007	−0.038	−0.108	−0.173
Stability of ambient light intensity	0.047	0.009	−0.078	0.006
Stability of number of incoming messages	−0.123*	−0.157*	−0.257**	−0.471**
Stability of phone unlocked periods	−0.091	−0.128	−0.268**	−0.373
Stability of call periods	−0.135**	−0.215***	−0.349***	−0.501**
Stability of number of outgoing messages	−0.104	−0.155*	−0.351***	−0.568***

Notes: $^{*}p < .05$, $^{**}p < .01$, $^{***}p < .001$

Supplementary Table 3: Correlations between the Stability Index and psychiatric symptom severity if computing the Stability Index over a 7-day window and requiring at least 4, 5, 6, or 7 "good data" days. These correlations hold across the entire one-year study period and for multiple participants (n $\geq$ 10). This table shows that even including people who only had 4 out of 7 days of sensing data available, we achieve significant correlations between our Stability Index and psychiatric symptom severity.
