# Supplementary material for: Assessing the relationship between routine and schizophrenia symptoms with passively sensed measures of behavioral stability
Source: NPJ Schizophr. 2020 Nov 23;6:35. doi: 10.1038/s41537-020-00123-2 (PMC7683525; doi:10.1038/s41537-020-00123-2)
Supplement: Supplementary file 2 — Reporting Summary [file 41537_2020_123_MOESM2_ESM.pdf]

nature research

Corresponding author(s): DBPR NPJSCHZ-00497

Last updated by author(s): Aug 31, 2020

Reporting Summary

Nature Research wishes to improve the reproducibility of the work that we publish. This form provides structure for consistency and transparency in reporting. For further information on Nature Research policies, see our Editorial Policies and the Editorial Policy Checklist.

Statistics

For all statistical analyses, confirm that the following items are present in the figure legend, table legend, main text, or Methods section.

n/a	Confirmed	
☐	☒	The exact sample size (*n*) for each experimental group/condition, given as a discrete number and unit of measurement
☐	☒	A statement on whether measurements were taken from distinct samples or whether the same sample was measured repeatedly
☐	☒	The statistical test(s) used AND whether they are one- or two-sided<br>*Only common tests should be described solely by name; describe more complex techniques in the Methods section.*
☒	☐	A description of all covariates tested
☒	☐	A description of any assumptions or corrections, such as tests of normality and adjustment for multiple comparisons
☒	☐	A full description of the statistical parameters including central tendency (e.g. means) or other basic estimates (e.g. regression coefficient) AND variation (e.g. standard deviation) or associated estimates of uncertainty (e.g. confidence intervals)
☒	☐	For null hypothesis testing, the test statistic (e.g. *F*, *t*, *r*) with confidence intervals, effect sizes, degrees of freedom and *P* value noted<br>*Give P values as exact values whenever suitable.*
☒	☐	For Bayesian analysis, information on the choice of priors and Markov chain Monte Carlo settings
☒	☐	For hierarchical and complex designs, identification of the appropriate level for tests and full reporting of outcomes
☐	☒	Estimates of effect sizes (e.g. Cohen's *d*, Pearson's *r*), indicating how they were calculated

Our web collection on statistics for biologists contains articles on many of the points above.

Software and code

Policy information about availability of computer code

Data collection	All participants were asked to carry a Samsung Galaxy S5 Android smartphone with CrossCheck pre-installed with them for 12 months. Three days per week, CrossCheck prompted participants to complete a brief self-report scale; at the same time, it collected data from passive sensors already installed on devices in the background of the user's otherwise routine device use.
Data analysis	The code that supports the findings of this study is publically available at: https://github.com/behavioral-data/stability-index

For manuscripts utilizing custom algorithms or software that are central to the research but not yet described in published literature, software must be made available to editors and reviewers. We strongly encourage code deposition in a community repository (e.g. GitHub). See the Nature Research guidelines for submitting code & software for further information.

Data

Policy information about availability of data

All manuscripts must include a data availability statement. This statement should provide the following information, where applicable:

- Accession codes, unique identifiers, or web links for publicly available datasets
- A list of figures that have associated raw data
- A description of any restrictions on data availability

The data that support the findings of this study will be publicly available, but it will happen in a separate process from this publication that has already started.

Field-specific reporting

Please select the one below that is the best fit for your research. If you are not sure, read the appropriate sections before making your selection.

☐ Life sciences ☒ Behavioural & social sciences ☐ Ecological, evolutionary & environmental sciences

For a reference copy of the document with all sections, see nature.com/documents/nr-reporting-summary-flat.pdf

Behavioural & social sciences study design

All studies must disclose on these points even when the disclosure is negative.

Study description	Data in this study were drawn from a randomized trial of mHealth monitoring intended to reduce psychiatric relapse in schizophrenia-spectrum disorders. Data in this report are from participants in the intervention group (i.e., CrossCheck condition). All participants in this condition were asked to carry a Samsung Galaxy S5 Android smartphone with CrossCheck pre-installed with them for 12 months. Three days per week, CrossCheck prompted participants to complete a brief self-report scale; at the same time, it collected data from passive sensors already installed on devices in the background of the user's otherwise routine device use. Data are quantitative.
Research sample	Participants were recruited from a large psychiatric hospital in New York. Participants included sixty-one (n = 61) adults with a schizophrenia-spectrum disorder and a recent (within past 12 months) significant psychiatric event, including either a psychiatric inpatient or daytime hospitalization, psychiatric ER visit, or outpatient crisis management. Exclusion criteria were: (1) sensory or physical impairments that would interfere with the use of a smartphone (determined via screening in vivo testing), (2) a < 6th grade reading level (per the Wide Range Achievement Test), or (3) lacking competency to consent to participate in research. Full descriptions of the study software and other studies examining CrossCheck data are available in this paper:<br><br>R. Wang, M. S. Aung, S. Abdullah, R. Brian, A. T. Campbell, T. Choudhury, M. Hauser, J. Kane, M. Merrill, E. A. Scherer, et al., "Crosscheck: toward passive sensing and detection of mental health changes in people with schizophrenia," in Proceedings of the 2016 ACM International Joint Conference on Pervasive and Ubiquitous Computing, pp. 886–897, ACM, 2016.
Sampling strategy	Participants included sixty-one (n = 61) adults with a schizophrenia-spectrum disorder and a recent (within past 12 months) significant psychiatric event, including either a psychiatric inpatient or daytime hospitalization, psychiatric ER visit, or outpatient crisis management. Exclusion criteria were: (1) sensory or physical impairments that would interfere with the use of a smartphone (determined via screening in vivo testing), (2) a < 6th grade reading level (per the Wide Range Achievement Test), or (3) lacking competency to consent to participate in research. Participants were recruited from a large psychiatric hospital in New York. Clinicians were asked by the research team to provide these prospective participants with a study description and post flyers. Study staff also reviewed electronic health records for potentially eligible clients to approach. The research team oriented potential participants to the study when these prospective participants authorized clinicians to share their contact information. After completion of written informed consent, participants were randomized into either: (1) the intervention group (i.e., with access to the CrossCheck system with as needed follow-up support), or (2) the treatment as usual group.
Data collection	Data in this report are from participants in the intervention group (i.e., CrossCheck condition). All participants in this condition were asked to carry a Samsung Galaxy S5 Android smartphone with CrossCheck pre-installed with them for 12 months. Three days per week, CrossCheck prompted participants to complete a brief self-report scale; at the same time, it collected data from passive sensors already installed on devices in the background of the user's otherwise routine device use. CrossCheck assessed physical activity using Google Activity Recognition Application Programming. Every ten seconds, CrossCheck generated a rating of which activity the participant engaged in or every 30 minutes when the device was held still. CrossCheck passively assessed (via the Smartphone microphone) the amount of time during which speech was present or near to the device, allowing for quantification of speech frequency (the number of discrete episodes during which the device detected speech) and speech duration (the summed length of these episodes over the course of a day). CrossCheck passively logged the number of SMS text messages sent and received as well as the number and duration of phone calls placed and received.
Timing	Data collection is from October 2015-October 2016
Data exclusions	Before calculating study variables, we filtered data to increase data quality. Consistent with previous work in our group, we included only periods wherein 7 "good data" days occurred during the previous 14-day period, with "good data" days defined as those in which more than 19 hours of sensing data were collected during that day. Since we needed at least 25 data points from each individual, we restricted further analyses to the 13 participants with more than 25 EMA responses over the entire study period (excluding 48 participants)
Non-participation	no participants dropped out
Randomization	After completion of written informed consent, participants were randomized into either: (1) the intervention group (i.e., with access to the CrossCheck system with as needed follow-up support), or (2) the treatment as usual group.

Reporting for specific materials, systems and methods

We require information from authors about some types of materials, experimental systems and methods used in many studies. Here, indicate whether each material, system or method listed is relevant to your study. If you are not sure if a list item applies to your research, read the appropriate section before selecting a response.

Materials & experimental systems

n/a	Involved in the study
☒	☐ Antibodies
☒	☐ Eukaryotic cell lines
☒	☐ Palaeontology and archaeology
☒	☐ Animals and other organisms
☐	☒ Human research participants
☐	☒ Clinical data
☒	☐ Dual use research of concern

Methods

n/a	Involved in the study
☒	☐ ChIP-seq
☒	☐ Flow cytometry
☒	☐ MRI-based neuroimaging

Human research participants

Policy information about studies involving human research participants

Population characteristics	Participants included sixty-one (n = 61) adults with a schizophrenia-spectrum disorder and a recent (within past 12 months) significant psychiatric event, including either a psychiatric inpatient or daytime hospitaliza- tion, psychiatric ER visit, or outpatient crisis management. Exclusion criteria were: (1) sensory or physical impairments that would interfere with the use of a smartphone (determined via screening in vivo testing), (2) a < 6th grade reading level (per the Wide Range Achievement Test), or (3) lacking competency to consent to participate in research.
Recruitment	Participants were recruited from a large psychiatric hospital in New York. Clinicians were asked by the research team to provide these prospective participants with a study description and post flyers. Study staff also reviewed electronic health records for potentially eligible clients to approach.
Ethics oversight	Data in this study were drawn from a randomized trial of mHealth monitoring intended to reduce psychiatric relapse in schizophrenia-spectrum disorders. This trial was approved by the IRBs of Dartmouth College (24356) and Northwell Health/ Long Island Jewish Medical Center (14-100B) and registered as a clinical trial (NCT01952041).

Note that full information on the approval of the study protocol must also be provided in the manuscript.

Clinical data

Policy information about clinical studies

All manuscripts should comply with the ICMJE guidelines for publication of clinical research and a completed CONSORT checklist must be included with all submissions.

Clinical trial registration	NCT01952041
Study protocol	The study protocol is not available due to patient privacy concerns, but might be available upon reasonable erquest.
Data collection	Data collection is from October 2015-October 2016. Data in this report are from participants in the intervention group (i.e., CrossCheck condition). All participants in this condition were asked to carry a Samsung Galaxy S5 Android smartphone with CrossCheck pre-installed with them for 12 months. Three days per week, CrossCheck prompted participants to complete a brief self-report scale; at the same time, it collected data from passive sensors already installed on devices in the background of the user's otherwise routine device use.
Outcomes	CrossCheck prompted participants to complete a 10-item self-report (EMA) questionnaire each Monday, Wednesday and Friday during the study period. This questionnaire began with the prompt, "Just checking in to see how you've been doing over the last few days." Table 3 shows the full list of EMA items; response options ranged from 0 (not at all) to 3 (extremely). For this analysis, we calculated overall EMA score as the sum of all negative items minus the sum of all positive items. This score ranges from −15 to 15, with a higher value suggesting greater symptom severity and poorer functioning.
